# Highly enriched exosomal lncRNA OIP5-AS1 regulates gastrointestinal stromal tumor (GIST) resistance to sunitinib through miR-145 and SOX9

**DOI:** 10.1080/15384047.2025.2522543

**Published:** 2025-06-23

**Authors:** Cui-Hua Wang, Xin-Ming Yao, Chun-Xia Pan, Hai-Feng Zhan, Hong-Feng Zhou

**Affiliations:** a Oncology Department, Shanghai Putuo District Liqun Hospital, Shanghai, China; b Oncology Department, The Third People’s Hospital of Dalian, Dalian, China; c Department of Gynecology, Jing An District Central Hospital, Shanghai, China; d Department of Medical Oncology, Harbin Medical University Cancer Hospital, Harbin, Heilongjiang, China

**Keywords:** Gastrointestinal stromal tumor, lncRNA, exosome, biomarker, drug resistance

## Abstract

Targeted therapy-induced resistance is a significant factor contributing to treatment failure in patients with gastrointestinal stromal tumors (GIST). Despite the identification of the long non-coding RNA (lncRNA) OIP5-AS1 as a critical player in human malignancy development, its role in GIST-related drug resistance remains largely unexplored. This study revealed substantial up-regulation of both OIP5-AS1 and SOX9, alongside significant down-regulation of miR-145, within sunitinib-resistant GIST cells. OIP5-AS1 emerged as a competing endogenous RNA, exerting inhibition on miR-145 while concurrently promoting the expression of SOX9. Exosome-mediated transfer of OIP5-AS1 induced heightened proliferation and invasion of GIST cells, culminating in the induction of chemoresistance to sunitinib through the miR-145/SOX9 axis. The knockdown of OIP5-AS1-expressing exosomes resulted in reduced cell proliferation and invasion in chemo-resistant GIST cells. In summary, these findings collectively suggest that OIP5-AS1 fosters GIST cell proliferation and invasion by suppressing miR-145 and up-regulating SOX9, ultimately contributing to drug resistance and tumor progression in GIST.

## 1. Introduction

Gastrointestinal stromal tumor (GIST) stands as the most prevalent mesenchymal tumor within the gastrointestinal tract.^1^ It has been observed that around 85% of GIST patients harbor activating mutations in either the receptor tyrosine kinase genes KIT or the platelet-derived growth factor receptor A (PDGFRA). This aberrant activation of KIT/PDGFRA initiates and advances GIST by triggering a sequence of downstream signaling pathways.^2^ Imatinib, a small molecule tyrosine kinase inhibitor (TKI), is effective in treating postoperative recurrence and metastasis of GIST, attributed to its disruption of KIT and PDGFRA signaling.^3^ As for Sunitinib, a second-generation KIT inhibitor, it plays a pivotal role in regulating both tumor angiogenesis and tumor cell proliferation. Sunitinib is approved for treating adults with imatinib-resistant GIST or those intolerant to imatinib. However, Sunitinib resistance presents a formidable obstacle in enhancing outcomes for GIST patients, with the precise underlying mechanisms remaining elusive.^4^

Long non-coding RNAs (lncRNAs) encompass non-protein-coding transcripts exceeding a length of 200 nucleotides (nt). The accruing evidence underscores lncRNAs’ involvement in diverse biological processes, such as cell growth, apoptosis, and differentiation.^5^ Recent studies have suggested an extensive role of lncRNAs in the initiation and progression of human malignancies, including GIST.^6^ The Prostate Cancer-Associated Transcript 6 (PCAT6) fosters GIST cell proliferation and stemness by functioning as a miR-143-3p/PRDX5 axis sponge.^7^ Likewise, HOTAIR activates autophagy, promoting GIST cell resistance to imatinib via the miR-130a/ATG2B pathway.^8^ In a recent study, the exosome-mediated transmission of OIP5-AS1 was demonstrated to engender trastuzumab resistance in breast cancer through the sequestration of miR-381-3p.^9^ OIP5-AS1‘s characterization as an oncogenic lncRNA is documented in various human malignancies, including renal, bladder, and lung cancers.^10^

Sox9 has been implicated in both the initiation and progression of several solid tumors. Its designation as a master regulator of morphogenesis during human development renders it an optimal candidate for disruption within malignant tissues.^11^ Emerging evidence has demonstrated that miR-145 targets the SOX9/ADAM17 axis, suppressing tumor-initiating cells and IL-6-mediated paracrine effects in head and neck cancer. Building on this, our bioinformatics analysis and *in vitro* experiments have newly identified SOX9 as a potential target of miR-145 in GIST cells. However, the regulatory relationship between miR-145 and SOX9, particularly their modulation by the long noncoding RNA OIP5-AS1, and their involvement in Sunitinib resistance in GIST cells, remains unexplored. This study seeks to address these critical gaps, providing novel insights into the miR-145/SOX9 axis and its potential role in overcoming therapeutic resistance in GIST.

In the meantime, OIP5-AS1 was forecasted to interact with miR-145. In light of these findings, our hypothesis revolves around the notion that the lncRNA OIP5-AS1 might play a pivotal role in gastrointestinal stromal tumor resistance to Sunitinib through the miR-145/SOX9 pathway. In this context, our aim is to scrutinize the functions of both OIP5-AS1 and the miR145/SOX9 axis concerning Sunitinib resistance, and additionally, to delve into the molecular mechanism underpinning targeted therapy-induced resistance in GIST.

## 2. Materials and methods

### 2.1. Cell culture

The parental Sunitinib-sensitive GIST-882 and GIST-T1 cell lines were obtained from the Cell Bank of the Type Culture Collection of the Chinese Academy of Sciences (Shanghai, China). The Sunitinib-resistant GIST-882 (GIST-882/Suni) and Sunitinib-resistant GIST-T1 cell lines were established through gradual exposure of the parent cells to increasing concentrations of Sunitinib.^12^ All cell lines were cultured in RPMI 1640 medium supplemented with 10% fetal bovine serum and maintained at 37°C in a humidified incubator with 5% CO_2_.

### 2.2. Cell transfection

To suppress OIP5-AS1 *in vitro* experiments, GIST-822 cells were transiently transfected with siRNA targeting OIP5-AS1 (si-OIP5-AS1, 30 nM, RiboBio, Guangzhou, China) or a negative control siRNA (si-NC) using Lipofectamine 3000 (Invitrogen) following the manufacturer’s instructions. For the generation of miR-145 overexpression and knockdown cells, GIST-822 cells were transfected with a miR-145 mimic, a miR-145 inhibitor (30 nM, RiboBio), or scrambled oligonucleotide sequences (miR-NC mimic or miR-inhibitor-NC) using Lipofectamine 3000. To achieve SOX9 knockdown in GIST-822 cells, transfection with 50 nM SOX9 siRNA (ON-TARGETplus Human SOX9 siRNA, no. L-021507-00–0005, Dharmacon) or negative control siRNA (ON-TARGETplus Non-Targeting Control Pool, no. D-001810-10–05, Dharmacon) was conducted using Lipofectamine 3000 Transfection Reagent (no. 11668027, Invitrogen) in Opti-MEM, following the manufacturer’s protocol.

### 2.3. ENCORI analysis

The ENCORI database (http://starbase.sysu.edu.cn/index.php) serves as an open platform for investigating interactions encompassing miRNA-ncRNA, miRNA-mRNA, ncRNA-RNA, RNA-RNA, RBP-ncRNA, and RBP- in CLIP-seq, degradome-seq, and RNA-RNA interaction group data. In this study, we employed the ENCORI database to confirm the associations involving OIP5-AS1 and miR-145, as well as SOX9 and miR-145.

### 2.4. Exosome isolation and labeling

Exosomes were isolated from GIST cell culture medium or serum samples (Table 1) using the ExoQuick precipitation kit (System Biosciences, LLC, Palo Alto, CA, USA). Briefly, samples were centrifuged at 3,000 × g for 15 minutes at 4°C to eliminate cell fragments. Subsequently, the centrifuged cell supernatant and exosome separation reagent were centrifuged at 10,000 g at 4°C for 2 hours. The exosome-containing pellet was then resuspended in 250 µl of PBS. Purified exosomes were labeled with the PKH26 Red Fluorescent Cell Linker kit (Sigma-Aldrich; Darmstadt, Germany) following the manufacturer’s instructions.

**Table 1.** Sequence of primers used in this study.

Genes	Forward/Reverse	Sequences
*SOX9*	Forward	5’-AGCGAACGCACATCAAGAC-3’
	Reverse	5’- CTGTAGGCGATCTGTTGGGG-3’
*miR-145*	Forward	5’-GTCCAGTTTTCCCAGGA-3’
	Reverse	5’- GAACATGTCTGCGTATCTC-3’
*OIPS-AS1*	Forward	5’-TGCGAAGATGGCGGAGTAAG-3’
	Reverse	5’- TAGTTCCTCTCCTCTGGCCG-3’
*ACTB*	Forward	5’-CACCATTGGCAATGAGCGGTTC-3’
	Reverse	5’-AGGTCTTTGCGGATGTCCACGT-3’
*U6*	Forward	5’-ATTGGAACGATACAGAGAAGATT-3’
	Reverse	5’-GGAACGCTTCACGAATTTG-3’

### 2.5. Characterization of exosomes

The purified exosomes underwent morphological characterization using transmission electron microscopy (TEM) (JEOL, Akishima, Japan). The purified exosome pellet was placed onto a carbon-coated copper grid, incubated for 5 minutes at 37°C, and subsequently immersed in a 2% phosphotungstic acid solution for 1 minute. Finally, TEM was employed to capture images of exosomes at a voltage of 100.0 kV and a magnification of × 60.0k. Furthermore, the sizes of the exosomes were assessed via nanoparticle tracking analysis (NTA) using the NanoSight NS300 system (NanoSight Technology, Malvern, UK). The results regarding exosome size were presented as a particle size distribution.

### 2.6. RNA extraction

RNAs were extracted from exosomes using the miRNeasy Serum/Plasma kit (Qiagen, Inc., Valencia, CA, USA). For RNA extraction from the cells, TRIZOL (Invitrogen; Thermo Fisher Scientific, Inc.) was employed. The purified RNA elusion was preserved in RNase-free ultra-pure water for subsequent use.

### 2.7. Reverse transcription-quantitative PCR (RT-qPCR)

Total RNAs were extracted from the collected cells using the TRIZOL reagent (Invitrogen, USA) and were subjected to reverse transcription reactions with the PrimeScript RT Reagent kit (Takara, Dalian, China). Subsequently, quantitative real-time PCR was performed using the fluorescent quantitative PCR kit (Takara, Dalian, China) on the ABI 7500 system (ABI, Foster City, CA, USA). U6 was chosen as the internal control for miRNA, and β-actin served as the endogenous control for mRNA. The equation 2^−ΔΔCt^ was utilized to determine the relative expression. The primer sequences for detecting the expression of SOX9, miR-145, OIP5-AS1, ACTB, and U6 are provided in Table 2.

**Table 2.** Baseline patients’ characteristics.

Patient characteristics	Sunitinib-resistant (n = 15)	Sunitinib-sensitive (n = 15)
Age (in years)		
Median	56	58
Range	26–70	30–69
Gender		
Male	10 (66.7)	8 (53.3)
Female	5 (33.3)	7 (46.7)
Co morbidities		
Diarrhea	5 (33.33%)	5(33.3%)
Hypertension	3 (20)	2 (13.3)
Debilitation	4(26.7%)	4(26.7%)
Hand-foot syndrome	2 (13.3)	2 (13.3)
Mucositis	2 (13.3)	3(20)
Past history of malignancy	1 (6.7)	0 (0)
Performance status		
0	2 (13.3)	2 (13.3)
1	8 (53.3)	8 (53.3)
2	4 (26.7)	4 (26.7)
3	1 (6.7)	1 (6.7)
Site of primary disease		
Stomach	5 (30)	5 (30)
Small intestine	7 (46.7)	7 (46.7)
Retroperitoneal	3(20)	3 (20)
Metastatic sites		
Liver metastasis	10 (66.7)	10 (66.7)
Node metastasis	5 (33.3)	5 (33.3)
Previous sunitinib		
Resistant	15 (100)	0 (0)
Intolerant	0 (0)	15 (100)
Starting sunitinib dose		
50 mg	15(100)	15 (100)
Duration of sunitinib therapy (in months)		
Median	23	23
Range	6–46	6–46

### 2.8. Dual-luciferase reporter gene assay

The sequences of the wild-type (WT) OIP5-AS1 and the sequences of the mutant type (MUT) resulting from site-directed mutation of the WT target site were cloned into the pmiR-RB-REPORT™ vector (RiboBio Co., Ltd., Guangzhou, China). The recombinant plasmids of MUT and WT OIP5-AS1 were co-transfected into 293T cells along with miR-NC or miR-145 mimic. After 48 hours, the relative luciferase activity was determined using firefly luciferase as an internal control. The sequences of WT and MUT OIP5-AS1 are as follows: OIP5-AS1-WT: 5’-AUCUUUUCAU – GAGAACUGGAA-3’, OIP5-AS1-MUT: 5’-AUCUUGCAAC – CUGCCUAUUGA-3’.

### 2.9. Cell counting kit-8 (CCK-8) assay

The CCK-8 Kit was employed following the manufacturer’s instructions (Beyotime, Shanghai, China). In short, cells (1000 cells/well) were seeded into the 96-well plates and then incubated for the specified time points. After 1, 2, 3, 4, and 5 days, CCK-8 reagents were introduced to each well, and the values of optical density (OD) were assessed using a microplate reader at 450 nm.

### 2.10. Colony formation assay

The transfected cells were seeded into 6-well plates (500 cell/well) for a colony formation assay and were cultured for 14 days. Subsequently, the medium was aspirated, and the cells were washed twice with PBS. The colonies were fixed using methanol and stained with 0.1% crystal violet. The rate of colony formation was determined using optical microscopy (Olympus, Tokyo, Japan).

### 2.11. Cell invasion assays

GIST-882 cells were seeded on the upper chamber of a 24-well Transwell membrane coated with Matrigel (BD Biosciences, San Jose, CA, USA) for the invasion assay. RPMI 1640 medium containing 10% FBS was introduced to the lower chambers. Following a 48-hour incubation at 37°C, cells on the upper membrane were detached. Subsequently, the cells were fixed with 4% paraformaldehyde, stained with 0.2% crystal violet, and quantified under an optical microscope (Olympus, Tokyo, Japan).

### 2.12. Western blot assay

Proteins from cells or exosomes were extracted using lysis buffer, and the protein concentration was determined using the BCA Protein Assay kit (Beyotime Institute of Biotechnology, China). Subsequently, protein lysates were subjected to 12% SDS-PAGE gels, transferred onto PVDF membranes (Millipore, Billerica, MA, USA), and then blocked with 5% nonfat milk. The membranes were incubated with primary antibodies against SOX9, CD63, TSG101, and β-actin (Abcam, Cambridge, MA, USA) overnight at 4 °C. Following this, the membranes were exposed to the corresponding secondary antibodies (Abcam, Cambridge, USA) at room temperature for 1 hour. Lastly, the membranes were visualized using the enhanced chemiluminescence (ECL) reagent (Pierce, Rockford, IL, USA) according to the manufacturer’s protocol.

### 2.13. In vivo study

Six-week-old male athymic BALB/c nude mice were maintained in a special pathogen-free (SPF) environment. GIST-882/Suni cells (transfected with siOIP5-AS1, siSOX9, and miR-145 mimic) were implanted under the armpits at a density of 5 × 10^7^ cells and allowed to grow for 4 weeks. Subsequently, the animals were randomly allocated into treatment groups each comprising five mice, for efficacy studies. Sunitinib (80 mg/kg/day) was administered daily via oral gavage. Tumor dimensions were captured using a Sony camera (Sony α-600). After 4 weeks, the mice were euthanized.

### 2.14. Statistical analysis

Data were presented as mean ± standard deviation (SD). Statistical analysis was conducted using GraphPad Prism 6.0 (GraphPad Software, San Diego, CA). Student’s T-test was employed to ascertain differences between two groups, while one-way analysis of variance (ANOVA) was utilized for the analysis of differences among three groups or more. The significance level of *p* < .05 was deemed statistically significant.

## 3. Results

### 3.1. Construction and analysis of the Sunitinib-resistant GIST-882/Suni cells in vitro and in vivo

**Figure 1.**
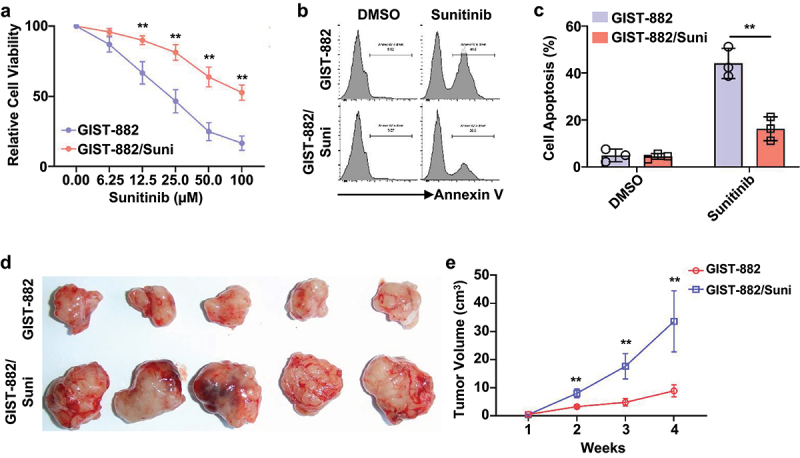
Construction and analysis of Sunitinib-resistant cells.

To further explore the resistance of GIST cells to Sunitinib treatment, both Sunitinib-sensitive GIST-882 cells and Sunitinib-resistant GIST-882/Suni cells were exposed to varying concentrations of Sunitinib for 24 hours. Then the cell viability and IC50 value for Sunitinib were determined by CCK-8 assay. We found that compared to those Sunitinib-sensitive GIST-882 cells, the Sunitinib-resistant GIST-882/Suni cells exhibited significantly augmented cell viability under different concentrations of Sunitinib exposure (Figure 1a). Additionally, a significantly high IC50 value of 98.5 μM for Sunitinib was observed in GIST-882/Suni cells compared to those GIST-882 cells (25 μM) (Figure 1a). Next, the results from cell apoptosis determination demonstrated that in comparison to the Sunitinib-sensitive GIST-882 cells, the Sunitinib-resistant GIST-882/Suni cells showed significantly low apoptotic rates after Sunitinib treatment (Figure 1b,c), suggesting significantly low sensitivity and high resistance of GIST-882/Suni cells to Sunitinib treatment *in vitro*. *In vivo* examinations further substantiated our *in vitro* results, revealing a conspicuous reduction in tumor volumes among mice hosting GIST-882/Suni cells following Sunitinib treatment. (Figure 1d,e).

(a) The viability of the Sunitinib-sensitive GIST-882 cells and Sunitinib-resistant GIST-882/Suni cells after different concentrations of Sunitinib treatment was monitored by CCK-8 method. (B, C) Representative histograms (b) and bar graph (c) showing the apoptotic rates of GIST-882 and GIST-882/Suni cells after different treatments. (d, e) Representative images (d) and bar graph (e) depicting the volume of tumors collected from the mice bearing GIST-882 or GIST-882/Suni cells after Sunitinib therapy (80 mg/kg/day). ** represents the p-value less than 0.01.

### 3.2. SOX9 is upregulated in resistant GIST cells and contributes to Sunitinib resistance

**Figure 2.**
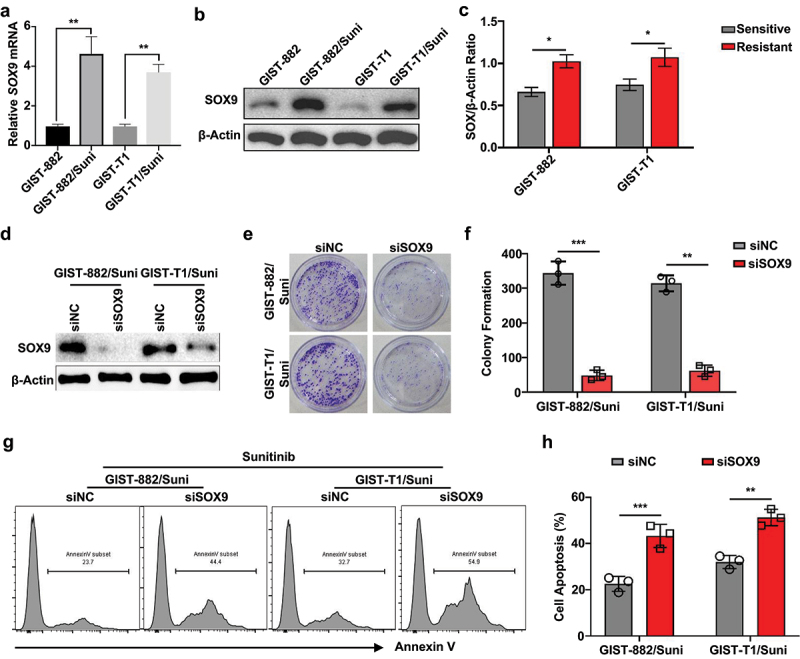
SOX9 is upregulated in Sunitinib-resistant GIST cells and promotes drug resistance.

Building upon our observation that Sunitinib-resistant GIST-882/Suni cells exhibit decreased apoptosis and elevated IC50 in response to Sunitinib (Figure 1), we next sought to identify potential drivers of this resistance. Given prior reports implicating SOX9 in oncogenic processes and drug resistance in other tumor types,^13^ We assessed its expression in both sensitive and resistant GIST cell lines. RT-qPCR analysis showed a significant increase in *SOX9* mRNA levels in GIST-882/Suni and GIST-T1/Suni cells compared to their parental counterparts (Figure 2a). Consistently, western blot analysis confirmed elevated SOX9 protein levels in the resistant cells (Figure 2b,c). Next, to determine the functional relevance of SOX9 in mediating drug resistance, we silenced SOX9 in GIST-882/Suni and GIST-T1/Suni cells using siRNA (Figure 2d). Colony formation assays revealed that SOX9 knockdown markedly impaired the clonogenic capacity of resistant cells under Sunitinib treatment (Figure 2e,f). Moreover, flow cytometric analysis demonstrated a significant increase in apoptotic cells upon SOX9 knockdown compared to control siRNA, in both resistant lines (Figure 2g,h). These results indicate that SOX9 upregulation plays a functional role in supporting the resistant phenotype of GIST cells, providing a rationale to further investigate its regulation.

(a) qRT-PCR analysis of *SOX9* mRNA levels in Sunitinib-sensitive (GIST-882, GIST-T1) and -resistant (GIST-882/Suni, GIST-T1/Suni) cells. (b, c) Western blot (b) and quantification (c) of SOX9 protein expression in Sunitinib-sensitive (GIST-882, GIST-T1) and -resistant (GIST-882/Suni, GIST-T1/Suni) cells. (d) Western blot confirming effective knockdown of SOX9 by siRNA in GIST-882/Suni and GIST-T1/Suni cells. (e, f) Representative images (e) and quantification (f) of colony formation assay in SOX9-silenced vs. control cells under Sunitinib treatment. (g, h) Flow cytometric analysis (g) and quantification (h) of apoptosis using Annexin V staining in SOX9-silenced vs. control GIST-882/Suni and GIST-T1/Suni cells treated with Sunitinib. *p < .05; **p < .01; ***p < .001.

### 3.3. Exosomal transfer of OIP5-AS1 from resistant cells enhances SOX9 expression and Sunitinib resistance in sensitive GIST cells

**Figure 3.**
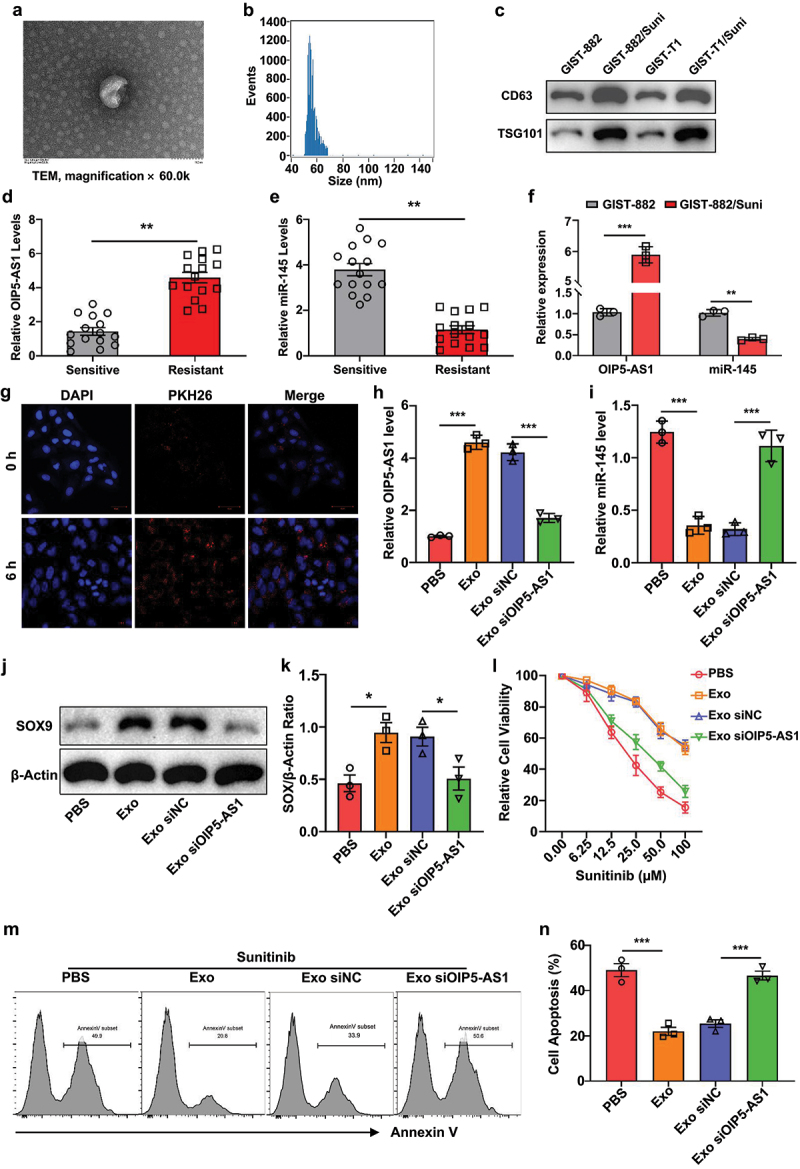
Exosomal transfer of OIP5-AS1 from resistant cells enhances SOX9 expression and Sunitinib resistance in sensitive GIST cells.

To investigate the role of exosome-mediated communication in GIST drug resistance, we first isolated exosomes from the culture supernatant of Sunitinib-resistant GIST cells. Transmission electron microscopy revealed that the isolated vehicles exhibited a typical cup-shaped morphology with a size around 100 nm (Figure 3a), which was further confirmed by nanoparticle tracking analysis (NTA) showing a size distribution ranging from 40–100 nm (Figure 3b). To verify the identity of the isolated vesicles, Western blot analysis was performed, revealing elevated levels of the exosomal markers CD63 and TSG101 in the purified exosome fractions (Figure 3c), confirming successful isolation of exosomes.

(a) Transmission electron microscopy (TEM) image showing the characteristic cup-shaped morphology of isolated exosomes (scale bar: 100 nm). (b) Nanoparticle tracking analysis (NTA) profile showing size distribution of exosomes, with a peak diameter between 40-100 nm. (c) Western blot analysis of exosomal markers CD63 and TSG101 in exosomes derived from GIST-882/Suni and GIST-T1/Suni cells. (d-e) RT-qPCR analysis of OIP5-AS1 (d) and miR-145 (e) expression in exosomes isolated from the serum of Sunitinib-sensitive and -resistant patients (*n* = 15 per group). (f) RT-qPCR analysis of OIP5-AS1 and miR-145 expression in GIST-882 and GIST-882/Suni cells. (g) Fluorescent imaging of PKH26-labeled exosomes (red) taken up by GIST-882 cells over time (0 h and 6 h). Nuclei were counterstained with DAPI (blue). (h-i) RT-qPCR analysis of OIP5-AS1 (h) and miR-145 (i) expression in GIST-882 cells treated with exosomes derived from GIST-882/Suni cells, with or without OIP5-AS1 knockdown. (j-k) Western blot (j) and quantification (k) of SOX9 protein levels in GIST-882 cells treated with PBS, exosomes, or OIP5-AS1-silenced exosomes. (l) Cell viability (CCK-8 assay) of GIST-882 cells exposed to Sunitinib under different exosome treatments. (m-n) Annexin V-based apoptosis assay (m) and quantification (n) in GIST-882 cells treated with exosomes with or without OIP5-AS1 knockdown and Sunitinib. *p < .05; **p < .01; ***p < .001.

We next compared the levels of OIP5-AS1 and miR-145 in exosomes derived from the serum of Sunitinib-resistant and -sensitive patients. RT-qPCR results revealed a significant increase in exosomal OIP5-AS1 and a concurrent decrease in miR-145 in the resistant group compared to the sensitive group (Figure 3d,e). Similarly, in Sunitinib-resistant GIST-882/Suni cells, the expression of OIP5-AS1 was markedly upregulated, while miR-145 was significantly downregulated, compared to parental GIST-882 cells (Figure 3f).

Subsequently, to confirm the uptake of exosomes by recipient cells, PKH26-labeled exosomes derived from GIST-882/Suni cells were co-cultured with GIST-882 cells. Confocal microscopy revealed prominent internalization of labeled exosomes after 6 hours of incubation (Figure 3g). Following this uptake, RT-qPCR analysis showed that exosome-treated GIST-882 cells exhibited significantly increased OIP5-AS1 expression and suppressed miR-145 levels compared to PBS-treated controls. Importantly, these effects were abolished when exosomes were derived from OIP5-AS1-silenced GIST-882/Suni cells (Figure 3h,i).

Additionally, to further determine the functional consequences of exosomal OIP5-AS1 transfer, we assessed SOX9 expression in recipient GIST-882 cells. Western blot analysis showed that treatment with exosomes from resistant cells induced SOX9 upregulation, whereas this effect was markedly attenuated when OIP5-AS1 was silenced in donor exosomes (Figure 3j,k). Functionally, exosomes from GIST-882/Suni cells significantly enhanced GIST-882 cell viability across a range of Sunitinib concentrations, while this enhancement was reversed by OIP5-AS1 knockdown in exosomes (Figure 3l). Furthermore, apoptosis assays demonstrated that exosomal OIP5-AS1 suppressed Sunitinib-induced apoptosis in GIST-882 cells, an effect reversed by exosomal OIP5-AS1 knockdown (Figure 3m,n). Collectively, these data support that OIP5-AS1 is delivered via exosomes from resistant to sensitive cells, where it downregulates miR-145, upregulates SOX9, and promotes Sunitinib resistance.

### 3.4. Chemo-resistant cell-derived exosomes promote GIST-882 cell growth and invasion

**Figure 4.**
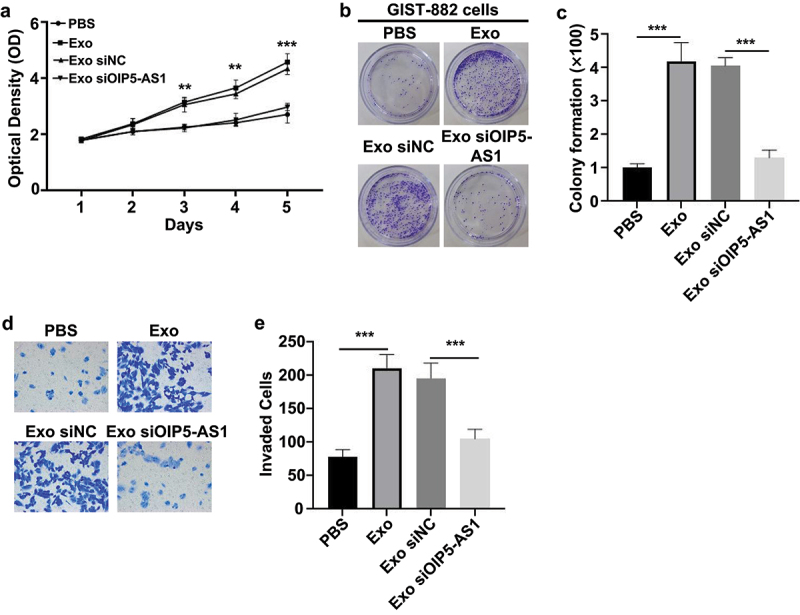
Chemo-resistant cell-derived exosomes promoted malignant phenotypes in the sensitive cells.

To further explore the role of exosomes derived from GIST-882/Suni cells in Sunitinib-sensitive GIST-882 cells, we monitored the growth and invasion of Sunitinib-sensitive GIST-882 cells following the specified treatments. As a result, in comparison to the cells co-cultured with PBS control, those co-cultured with exosomes from GIST-882/Suni cells displayed notably enhanced proliferation (Figure 4a), colony formation (Figure 4b,c), and invasion (Figure 4d,e). However, OIP5-AS1 knockdown in exosomes reversed the drug-resistant phenotype induced by exosome transfer from resistant cells (Figure 4a–e). These findings demonstrate that exosomes from Sunitinib-resistant cells can promote the proliferation, colony formation, and invasion of Sunitinib-sensitive GIST-882 cells under Sunitinib exposure, and this promotion is significantly dependent on the presence of OIP5-AS1 in exosomes.

(a) The CCK-8 assay was employed to measure the proliferation of the cells after differential treatments. (b and c) Representative images (b) and bar graph (c) depict the colony formation of GIST-882 cells after indicated treatments (500 cell/well). (d and e) Representative images (d) and bar graph (e) show the invasive potential of GIST-882 cells after indicated treatments. **p < .01; ***p < .001.

### 3.5. OIP5-AS1 promotes Sunitinib resistance by modulating the miR-145/SOX9 axis

**Figure 5.**
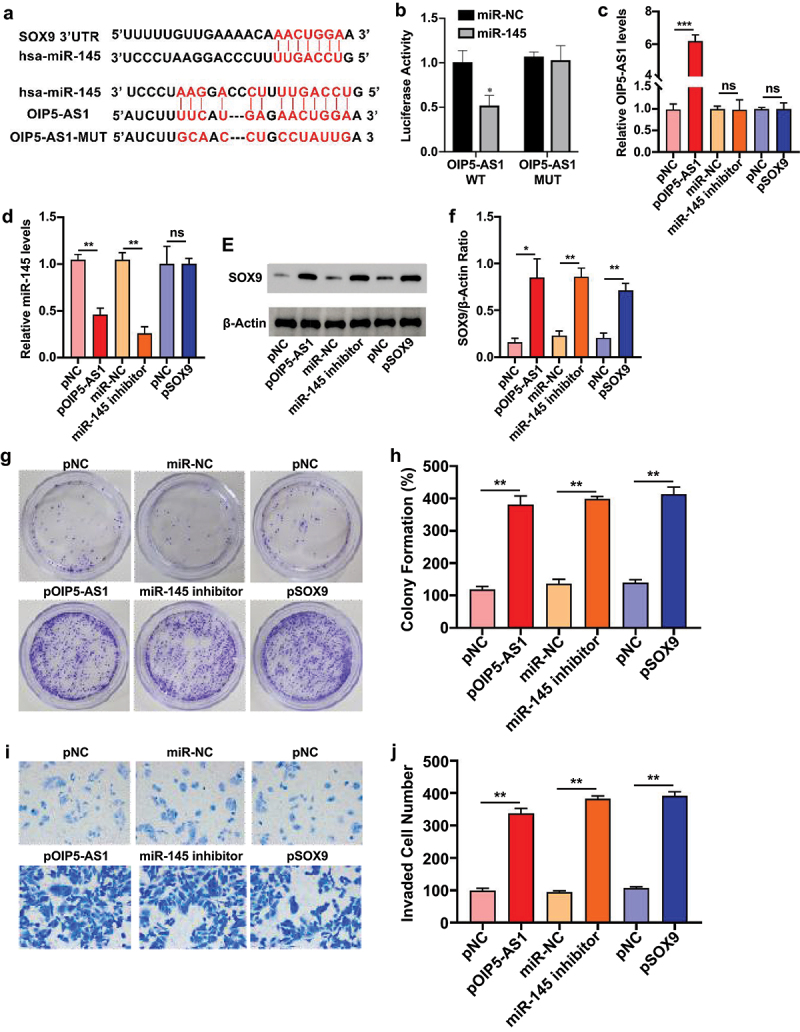
Elevated OIP5-AS1, SOX9, and suppressed miR-145 enhanced the chemosensitivity in GIST1882 cells.

To further dissect the regulatory relationship between OIP5-AS1, miR-145, and SOX9, we conducted analysis using the ENCORI database. Predicted binding sites indicated that miR-145 could directly target both the 3′UTR of SOX9 mRNA and OIP5-AS1 (Figure 5a). Dual-luciferase reporter assays confirmed that miR-145 significantly suppressed the luciferase activity of wild-type OIP5-AS1 (OIP5-AS1-WT) but not that of the mutant construct (OIP5-AS1-MUT), confirming a direct interaction between OIP5-AS1 and miR-145 (Figure 5b).

(a) Bioinformatic analysis of putative binding regions between SOX9 and miR-145, and OIP5-AS1 and miR-145. (b) The dual-luciferase reporter assay was employed in GIST-882 cells to determine the interaction between OIP5-AS1 and miR-145. (c and d) Representative bar graphs depict the transcription of OIP5-AS1 (c) and miR-145 (d) in the cells transfected with OIP5-AS1, SOX9 overexpressing plasmids, miR-145 inhibitors, and their negative controls (pNC or miR-NC). (e and f) Representative blots (e) and bar graph (f) show the translation of SOX9 in differentially treated cells. (g-j) Representative images (g and i) and bar graphs (h and j) represent the colony formation (g and h) and invasive potential (i and j) of differentially treated cells exposed to Sunitinib treatment. *p < .05. **p < .01. ***p < .001.

Subsequently, we evaluated the effect of OIP5-AS1 overexpression, SOX9 overexpression, and miR-145 inhibition on the expression of key axis components in GIST-882 cells. As shown in Figure 5c–f, overexpression of OIP5-AS1 significantly decreased miR-145 levels and elevated SOX9 expression at the protein level. Inhibition of miR-145 similarly upregulated SOX9 but did not affect OIP5-AS1 levels. Conversely, overexpression of SOX9 did not alter either OIP5-AS1 or miR-145 expression. These results demonstrate that OIP5-AS1 acts upstream of miR-145 and SOX9, promoting SOX9 expression via miR-145 suppression. Functionally, OIP5-AS1 overexpression, miR-145 inhibition, or SOX9 overexpression significantly enhanced the clonogenicity (Figure 5g,h) and invasive capacity (Figure 5i,j) of GIST-882 cells under Sunitinib exposure, further indicating that this axis contributes to chemoresistance.

**Figure 6.**
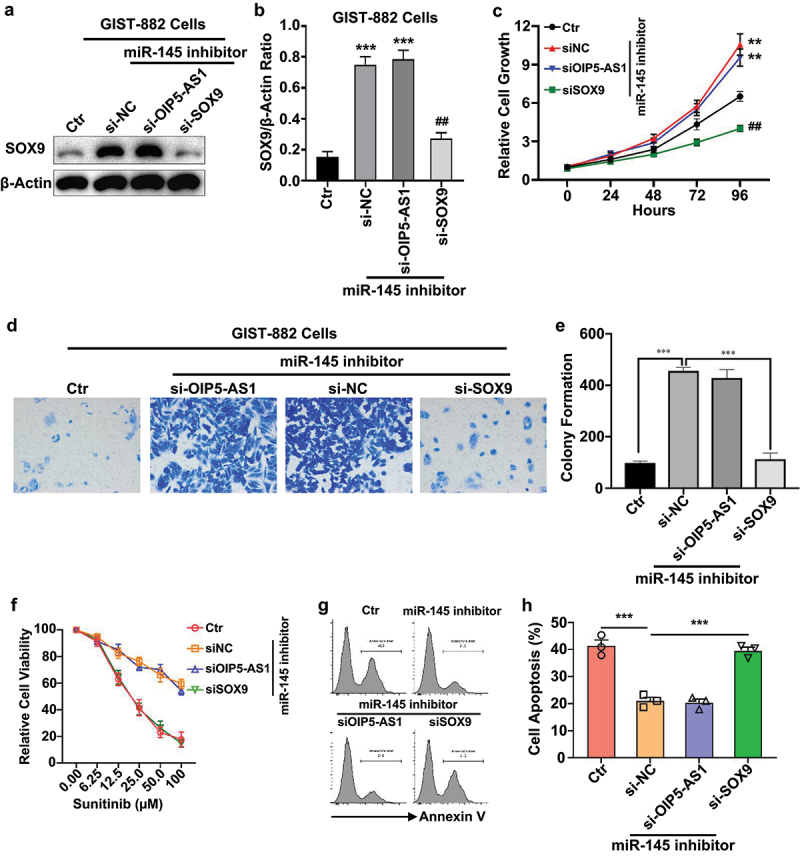
OIP5-AS1 targeted miR-145/SOX9 axis and regulated chemosensitivity of GIST-882 cells.

Additionally, to confirm this regulatory axis in a loss-of-function setting, we next silenced OIP5-AS1 or SOX9 in the context of miR-145 inhibition. Western blot analysis showed that knockdown of OIP5-AS1 or SOX9 decreased SOX9 protein levels even in the presence of miR-145 inhibitor (Figure 6a,b), suggesting that SOX9 expression requires both suppression of miR-145 and support from OIP5-AS1. Consistently, cell growth curves (Figure 6c) and colony formation (Figure 6d,e) demonstrated that the miR-145 inhibitor-induced increase in aggressiveness and drug resistance was abrogated by simultaneous knockdown of OIP5-AS1 or SOX9. Moreover, CCK-8 viability assays showed that cells with miR-145 inhibition alone exhibited increased resistance to Sunitinib, while co-silencing of OIP5-AS1 or SOX9 significantly restored drug sensitivity (Figure 6f). Apoptosis analysis further confirmed these findings, with the combination of SOX9 or OIP5-AS1 knockdown and miR-145 inhibition leading to increased Annexin V-positive cells (Figure 6g,h). Together, these data confirm that OIP5-AS1 confers Sunitinib resistance by downregulating miR-145 and subsequently upregulating SOX9. Targeting this regulatory axis may offer a promising strategy to overcome therapeutic resistance in GIST.

GIST-882 cells were treated with miR-145 inhibitor, then transfected with siOIP5-AS1 or siSOX9, or negative control (si-NC). (a and b) Representative blots (a) and bar graph (b) depict the expression of SOX9 in differentially treated cells. ***, *p* < .001 compared to the Ctr group. ##, *p* < .01 compared to the si-NC group. (c) Representative Curves shows the growth of cells after indicated treatments. **, *p* < .01 compared to the Ctr group. ##, *p* < .01 compared to the si-NC group. (d and e) Representative images (d) and bar graph (e) show the colony formation of differentially treated cells. ***, *p* < .001. (f) The viability of GIST-882 cells after indicated treatments. (g, h) Representative histograms (g) and bar graph (h) showing the apoptotic rates of GIST-882 cells after indicated treatments. *p < .05. **p < .01. ***p < .001.

### 3.6. Silencing of OIP5-AS1 increased Sunitinib sensitivity

**Figure 7.**
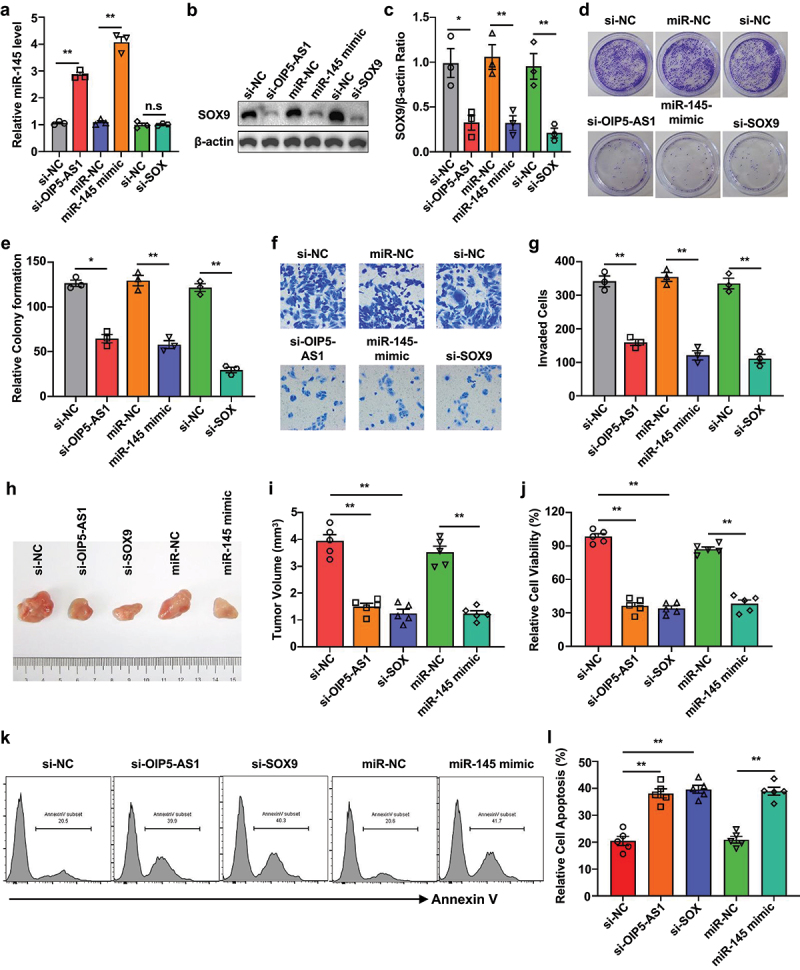
Exosome-delivered si-OIP5-AS1 increased sunitinib sensitivity.

Given the understanding that exosomes confer chemoresistance in sensitive cells, we sought to determine whether the suppression of the OIP5-AS1/miR-145/SOX9 axis could potentially reverse drug resistance in GIST cells. In GIST-882/Suni cells, siRNA targeting OIP5-AS1 and SOX9 was employed to inhibit the expression of OIP5-AS1 and SOX9, while the miR-145 mimic was used to elevate the levels of miR-145. As depicted in Figure 7a, the level of miR-145 was increased by si-OIP5-AS1 or the miR-145 mimic, but not by the suppression of SOX9 (Figure 7a). Additionally, SOX9 protein expression was also assessed using Western blot analysis, and our data revealed that the expression of SOX9 in cells transfected with si-OIP5-AS1, miR-145 mimic, or si-SOX9 is significantly suppressed compared to their respective negative controls (NC) (Figure 7b,c). As expected, a dramatic reduction in colony formation (Figure 7d,e) and cell invasion (Figure 7f,g) of GIST-882/Suni cells was observed after suppressing the expression of OIP5-AS1 and SOX9, as well as enhancing the expression of miR-145, indicating the significant regulatory role of the OIP5-AS1/miR-145/SOX9 axis in the sensitivity of GIST-882 cells to Sunitinib. Furthermore, GIST-882/Suni cells were inoculated into 6-week-old nude mice under the armpits and then treated with Sunitinib (80 mg/kg/day). As shown in Figure 7h,i, significantly smaller tumor volumes were observed in the mice from the siOIP5-AS1, siSOX9, and miR-145 mimic groups compared to the negative controls. This observation was also supported by the results obtained from cell viability (Figure 7j) and apoptosis (Figure 7k,l) examinations. This data suggests that targeting the OIP5-AS1/miR-145/SOX9 axis represents a novel strategy to enhance drug sensitivity in GIST.

(a) RT-qPCR analysis of the expression levels of miR-145 in GIST-882/Suni cells transfected with OIP5-AS1 siRNA, miR-145 mimic, and siSOX9. (b and c) GIST-882/Suni cells were transfected with siRNA for OIP5-AS1 and SOX9, and miR-145 mimic. Subsequently, western blot analysis was performed to determine the expression levels of SOX9 in each group. Representative blots (b) and bar graph (c) showing the expression of SOX9 in differentially treated cells. (d-g) Determination of the colony formation (d and e) and invasive (f and g) potential of the cells after differential treatments. (h and i) Mice were subcutaneously injected with GIST-882/Suni cells transfected with siOIP5-AS1, siSOX9, or miR-145 mimic. The tumor volume was monitored (*n* = 5). * *p* < .05; ** *p* < .01. n.s. not significant. (j) The viability of GIST-882/Suni cells with indicated treatments was monitored using CCK-8 assay. (k, l) Representative histograms (k) and bar graph (L) showing the apoptotic rates of GIST-882/Suni cells after indicated treatments. *p < .05. **p < .01. ***p < .001.

## 4. Discussion

This study demonstrates that the lncRNA OIP5-AS1 enriched from exosomes plays a pivotal role in conferring resistance to Sunitinib in GIST tumor cells. Analyzing the mechanisms underlying the regulatory effect of OIP5-AS1 on GIST cell resistance to Sunitinib, our primary findings can be summarized as follows: (1) Exosomes derived from chemo-resistant cells significantly diminish the sensitivity of tumor cells to Sunitinib treatment. (2) The induction of Sunitinib resistance in tumor cells by exosomes is closely governed by the miR-145/SOX9 axis. (3) OIP5-AS1 binds to miR-145 and modulates its expression in Sunitinib-resistant tumor cells. (4) Exosomes from chemo-resistant cells instruct GIST cells to overcome Sunitinib treatment through the OIP5-AS1/miR-145/SOX9 axis. Consequently, OIP5-AS1 enrichment within exosomes promotes the proliferation and invasion of GIST cells, enabling them to overcome Sunitinib treatment.

While targeted drugs and immunotherapy have significantly improved the prognosis of cancer patients and advanced anti-tumor treatment methods in recent years, drug resistance to targeted therapies and immunotherapy remains an unavoidable challenge. GIST, the most prevalent mesenchymal tumors in the alimentary tract, are typically managed with tyrosine kinase inhibitors (TKIs). Nevertheless, resistance to imatinib or sunitinib poses a substantial hurdle to effective GIST treatment. Thus, enhanced comprehension of molecular oncogenesis and mechanisms of drug resistance has opened new avenues for GIST treatment. Long non-coding RNAs (lncRNAs) are implicated in various biological behaviors, including the regulation of GIST.^8^ In this study, we focus on investigating the role of lncRNAs to uncover the molecular mechanisms underlying chemoresistance in GIST.

The lncRNA OIP5-AS1 is up-regulated in several human cancers and contributes to oncogenic transformation.^14,15^ Studies have demonstrated that OIP5-AS1 is up-regulated in pancreatic ductal adenocarcinoma (PDAC), and a high level of OIP5-AS1 predicts a poor prognosis in PDAC patients.^16^ Additionally, OIP5-AS1 is frequently elevated in breast cancer tumor tissues; inhibition of OIP5-AS1 suppresses proliferation, migration, and invasion of breast cancer cells,^17^ which is consistent with the inhibition of OIP5-AS1 that suppresses proliferation, aggressiveness, and induces cell cycle arrest and apoptosis in HCC cells.^18^

In our study, OIP5-AS1 was significantly elevated (Figure 3a), while miR-145 expression was decreased in sunitinib-resistant GIST patients (Figure 3a,b), indicating that the lncRNA OIP5-AS1 could play a crucial role in GIST. OIP5-AS1 was up-regulated in GIST-882 cells resistant to sunitinib (Figure 3c). A previous study has shown that exosome-mediated transfer of OIP5-AS1 promoted chemoresistance via sponging the miR-381-3p/HMGB3 axis in breast cancer cells.^11^ Exosomes can be selectively taken up by cells and can transfer cargos to reprogram the recipient cells with their bioactive compounds. Previous studies have shown that exosomes can be endocytosed by many mammalian cells, such as H9C2 cells,^19^ IEC-6 cells,^20^ HucMSCs.^21^ However, there were no studies reporting that exosomes were endocytosed into GIST-882 cells. Our study was the first to demonstrate that PKH26-labeled exosomes can be endocytosed into GIST-882 cells (Figure 3d). Currently, our findings showed that exosomes from Sunitinib-resistant cells transfer OIP5-AS1 into the recipient cells, suppressing miR-145 and up-regulating SOX9 in GIST cells. Our results suggested that highly enriched exosomal lncRNA OIP5-AS1 could induce drug resistance in GIST cells via the miR-145/SOX9 axis. Conversely, the suppression of OIP5-AS1 or SOX9 attenuated cell proliferation and invasion in the chemo-resistant GIST cells (Figure 4). However, suppressing OIP5-AS1 in miR-145 inhibitor-treated GIST-882 cells cannot attenuate cell proliferation (Figure 6). Collectively, these findings demonstrated that OIP5-AS1 contributes to drug resistance via the miR-145/SOX9 axis in sensitive GIST cells.

In addition, studies have demonstrated that miR-145 could attenuate chemoresistance and suppress tumor progression,^22^ which has been widely reported to function as a tumor suppressor in multiple human cancers.^23,24^ High levels of SOX9 promote tumor cell proliferation in vitro and in vivo using different malignant models, including gastric cancer, glioblastoma, and pancreatic adenocarcinoma.^25^ Thus, inhibition of SOX9 has been proposed as an effective strategy against gastrointestinal cancer. Our study found that resistant GIST cells deliver OIP5-AS1 to the sensitive cancer cells through exosomes, promoting drug resistance and tumor progression in GIST; our further study demonstrated that OIP5-AS1 could facilitate the expression of SOX9 by suppressing miR-145 expression.

Additionally, the location of OPI5-AS1 is significantly linked to its functions in different types of cells. Several publications have reported the location of OIP5-AS1 in various cell types. For example, in the publication by Dr. Bai Y et al.,^26^ it was observed that OIP5-AS1 is mostly located in the nucleus of gastric cancer cells, thus potentially epigenetically silencing NLRP6 expression through its interaction with EZH2. Meanwhile, the study conducted by Dr. Lili Han et al.^27^ demonstrated that OIP5-AS1 is primarily localized in the cytoplasm of cervical cancer cells and regulates the Warburg effect through the miR-124-5p/IDH2/HIF-1a pathway. Furthermore, in two other studies,^28,29^ the authors illustrated that OIP5-AS1 could contribute to the development of endometrial carcinoma cells by targeting miR-152-3p to up-regulate SLC7A5 and promote breast cancer metastasis via the miR-340-5p/ZEB2 axis, respectively. Both of these groups also observed that most of OIP5-AS1 is located in the cytoplasm. Therefore, based on these published findings, conclusions, and our current results, we speculate that OIP5-AS1 is primarily located in the cytoplasm of gastrointestinal stromal tumor cells.

## 5. Conclusion

Our study suggests that a high level of OIP5-AS1 is positively correlated with SOX9 activation, which may unveil a novel strategy to enhance drug sensitivity in GIST by targeting the OIP5-AS1/miR-145/SOX9 axis.

## Data Availability

Additional data to that included in the manuscript can be provided under request.
